# Editorial: Oral health and successful aging: integrating basic research, clinical insights and community health

**DOI:** 10.3389/fdmed.2026.1954839

**Published:** 2026-09-03

**Authors:** Jonathan Y. An, Maria Cecilia Ciaccio Vendola, Wilson Jacob-Filho, Martha J. Somerman

**Affiliations:** 1 Department of Oral Health Sciences, School of Dentistry, University of Washington, Seattle, WA, United States; 2 Division of Geriatrics, Department of Internal Medicine, University of São Paulo Medical School, São Paulo, Brazil; 3 Frontiers in Dental Medicine, Bethesda, MD, United States

**Keywords:** cellular senescence, geriatric dentistry, integrated care, older adults, oral frailty, oral health, successful aging

## Introduction

Healthy aging is not simply the absence of disease. The World Health Organization defines it as maintaining the functional ability that enables people to do what they value. That ability reflects intrinsic capacity—the combined physical and mental capacities an individual can draw on—together with the environment in which that person lives (1, 2). Oral health belongs within this framework. Although it is not currently a separate intrinsic-capacity domain, dentition and prostheses, periodontal health, salivary function, chewing, and swallowing influence nutritional reserve, communication, social participation, and independence. The relationship is bidirectional: cognitive decline, reduced mobility and dexterity, multimorbidity, polypharmacy, and limited access to care can rapidly erode oral hygiene and oral function. From a dental perspective, oral frailty is best understood as loss of oral function and reserve, not simply the presence of disease (3, 4). This Research Topic includes 10 articles: four Original Research articles, one Brief Research Report, four Systematic Reviews, and one Mini Review.

## Oral frailty, intrinsic capacity, and clinical function

Four systematic reviews establish oral frailty as a clinically relevant but still inconsistently defined construct. J. Huang et al. linked functional dentition, oral hygiene behaviors, and occlusal force with physical frailty. P. Huang et al. identified age, sex, physical frailty or pre-frailty, and unpartnered status as correlates. Zhu et al. documented studies for prevalence and these associations with unfavorable outcomes, while Dou et al. estimated that more than one-third of community-dwelling older adults are affected. The common lesson is that tooth count alone is insufficient. Oral function depends on occlusal support, prosthesis use and fit, salivary status, tongue strength, chewing, swallowing, and the practical capacity to perform daily care.

The intrinsic-capacity framework helps place these findings in context. Oral frailty should not simply be declared a sixth domain; it is better viewed as an oral-functional phenotype that intersects with several domains—particularly vitality, cognition, psychological capacity, and locomotion—and with environmental supports for eating and self-care. In a frailty-clinic cohort, a greater number of intrinsic-capacity deficits was associated with oral hypofunction while recent community-based data linked cumulative oral deficits with intrinsic-capacity deficits (5, 6). These cross-sectional findings support inclusion of focused oral assessment in person-centered geriatric care.

Xu et al. add behavioral and nutritional context: among older adults with hypertension, anxiety and nutritional status partly mediated the association between self-management ability and oral frailty. Song et al., using network analysis in rural China, identified connections among oral health, cognition, and activities of daily living, with sex-specific patterns. Both studies reinforce that oral vulnerability rarely occurs in isolation.

## From detection to function-preserving care

Meyer-Hofmann et al. evaluated calprotectin and active matrix metalloproteinase-8 in gingival crevicular fluid in geriatric inpatients. The study illustrates the potential value of low-burden biomarkers in care-dependent populations, while appropriately leaving clinical thresholds and external validation to larger studies. Yumol et al., using the Canadian Longitudinal Study on Aging, found no association between tooth number and hip bone mineral density in the combined sample. That negative result is informative as the number of teeth is easy to record, but does not inform oral health history.

Abe et al. reported that older adults with fewer than 20 teeth who did not use dentures had a greater increase in urinary sodium-to-potassium ratio than those using dentures. This does not prove that dentures improve diet, but that prosthesis quality, comfort, occlusal stability, and actual use matter. Nonetheless, it raises a question familiar to prosthodontic practice: does restoring a stable, functional dentition preserve dietary variety and nutritional reserve? Okubo et al. extend that question to home-based care, where dysphagia and heart failure can reinforce malnutrition, sarcopenia, aspiration risk, and hospital readmission. Their review makes a strong case for coordinated management among dentists, physicians, nurses, dietitians, and speech-language pathologists.

For clinicians, the practical message is to assess function, not only disease. Periodontal inflammation, pain, xerostomia, prosthesis fit, occlusal support, oral hygiene capacity, chewing, swallowing, cognition, nutrition, and access to care belong in the same evaluation. The purpose is not merely to label decline, but to identify what remains reversible and preserve eating, speaking, comfort, and autonomy.

## Linking oral aging to geroscience

Clinical function also needs a biological foundation. Salivary gland hypofunction, impaired periodontal repair, alveolar bone loss, altered mucosal immunity, and oral dysbiosis share features with aging in other tissues, including cellular senescence, chronic low-grade inflammation, and impaired regeneration (7, 8). Yet oral tissues remain underrepresented in major aging initiatives. This is a missed opportunity. Saliva, gingival crevicular fluid, exfoliated epithelial cells, and, when clinically indicated, gingival tissue can be sampled repeatedly. Incorporating oral tissues into efforts such as the Cellular Senescence Network and oral outcomes into aging trials would connect mechanistic discovery with outcomes that matter to patients (9).

Geroscience may clarify mechanisms and identify future targets, but its promise for oral health will ultimately be tested in the clinic. The near-term priority is to define which oral cell populations change with age, how those changes differ from active disease, and whether they predict impaired healing or loss of function. Periodontal disease may offer a natural proving ground: it is common in older adults, shares core features with aging elsewhere in the body—chronic inflammation, impaired regeneration, and microbial dysbiosis—and is readily monitored through accessible tissues and fluids. Preclinical work suggests that interventions targeting fundamental aging processes can attenuate, and perhaps partly reverse, age-associated periodontal breakdown, providing a rationale for carefully designed intervention trials that pair validated oral endpoints with markers of biological aging. Such trials would test whether modifying the biology of aging preserves oral function, while embedding oral outcomes in the broader effort to extend health span. This work would strengthen established preventive, periodontal, restorative, and prosthodontic care.

## Conclusion

Taken together, these articles underscore that oral health is part of the functional capacity that makes successful aging possible. Progress requires standardized oral-frailty measures, longitudinal studies, and care pathways that recognize oral health care as an essential component of whole-person health.
